# Patient with flank pain and intermittent hematuria. The typical renal colic? Usefulness of ultrasonography in the emergency room

**DOI:** 10.1186/2036-7902-7-S1-A16

**Published:** 2015-03-09

**Authors:** AA Oviedo-García, M Algaba-Montes

**Affiliations:** 1 Emergency Department, Valme Hospital, Seville, Spain

## Background

Acute flank pain and hematuria is a common ED presenting symptom, Bedside emergency renal US performed and interpreted by EPs with limited training and experience is increasing in use and gaining acceptance. Emergency renal ultrasonography concentrates on the focused presence or absence of hydronephrosis as is often seen in patients with acute flank pain secondary to renal colic. ED visit rates for urolithiasis increased from 178 to 340 visits per 100,000 individuals from 1992 to 2009. Therefore, it is a common condition in the ED. In many patients, bedside renal US may obviate the need for further diagnostic workup and speed the diagnosis and treatment of an emergency patient.

## Objective

We present a case of patient admited at ER with right flank pain and hematuria, the tipical presentation of renal colic.

## Patients and methods

renal calculi are the most common cause of flank pain and hematuria, it is prudent to also closely examine the kidneys on bedside emergency ultrasound for abnormal findings beyond the mere presence or absence of hydronephrosis.

## Results

53 year old male, was admitted to the ER by right flank pain and hematuria. Bedside emergency US initially performed to look for hydronephrosis, showed a large right renal mass, and and prompted further workup with CT of abdomen and pelvis. While US is less sensitive than CT for detecting renal masses, it is a convenient imaging modality with many potential benefits for the inicial ED workup of flank pain and hematuria.

## Conclusion

In this case study, emergency ultrasound helped to identify a renal mass in a patient who presented with hematuria and left flank pain, initially thought to be renal colic on clinical evaluation. Like most renal tumors, this patients symptoms overlapped with the typical presentation of renal calculi. It was the findings on emergency ultrasound, performed and interpreted by EPs, that helped to identify the correct diagnosis and prompted the appropriate consultations to urologist, with a final diagnostic of Renal Cell Carcinoma.

## Informed consent

The study was conducted in accordance with the ethical standards dictated by applicable law. Informed consent was obtained from each owner to enrolment in the study and to the inclusion in this article of information that could potentially lead to their identification.
